# Visualizing Meta-Features in Proteomic Maps

**DOI:** 10.1186/1471-2105-12-308

**Published:** 2011-07-28

**Authors:** Eugenia G Giannopoulou, George Lepouras, Elias S Manolakos

**Affiliations:** 1 HRH Prince Alwaleed Bin Talal Bin Abdulaziz Alsaud Institute for Computational Biomedicine, Weill Cornell Medical College, 1305 York Avenue, New York, NY, 10021, USA; 2 Department of Computer Science and Technology, University of Peloponnese, Tripolis, Greece; 3 Department of Informatics and Telecommunications, University of Athens, Panepistimiopolis, Athens, Greece

## Abstract

**Background:**

The steps of a high-throughput proteomics experiment include the separation, differential expression and mass spectrometry-based identification of proteins. However, the last and more challenging step is inferring the biological role of the identified proteins through their association with interaction networks, biological pathways, analysis of the effect of post-translational modifications, and other protein-related information.

**Results:**

In this paper, we present an integrative visualization methodology that allows combining experimentally produced proteomic features with protein meta-features, typically coming from meta-analysis tools and databases, in synthetic Proteomic Feature Maps. Using three proteomics analysis scenarios, we show that the proposed visualization approach is effective in filtering, navigating and interacting with the proteomics data in order to address visually challenging biological questions. The novelty of our approach lies in the ease of integration of any user-defined proteomic features in easy-to-comprehend visual representations that resemble the familiar 2D-gel images, and can be adapted to the user's needs. The main capabilities of the developed VIP software, which implements the presented visualization methodology, are also highlighted and discussed.

**Conclusions:**

By using this visualization and the associated VIP software, researchers can explore a complex heterogeneous proteomics dataset from different perspectives in order to address visually important biological queries and formulate new hypotheses for further investigation. VIP is freely available at http://pelopas.uop.gr/~egian/VIP/index.html.

## Background

The objective of large-scale proteomics analyses is to study the expression, function, modifications and interactions of proteins, and thus provide answers to challenging biological questions [1-4]. High-throughput proteomics techniques include several experimental steps (e.g., 2D Gel Electrophoresis-2DGE, Liquid Chromatography-LC, Mass Spectrometry-MS) that produce large volumes of data [4-6]. Meta-analysis follows and enriches the pool of proteomic features [7] with metadata, such as Gene Ontology (GO) annotation, information about networks, pathways, and more. In biomarker discovery studies in particular, it is necessary to integrate experimental results with metadata coming from various databases, pathway analysis software, and other sources, in order to identify biologically relevant biomarkers [8-13]. Information visualization techniques have become a powerful tool for bioinformatics and systems biology applications, since they help address the inherent difficulties in understanding large volumes of heterogeneous data [14-16]. Visualization methods assist in exploring the experimental results more efficiently than by simply examining numbers in large-size tables and lists [15,16], which lack the spatial organization and conceal the relative quantification aspects that the human eye can easily recognize. The necessity to manage diverse proteomics data and combine them in order to facilitate the interpretation of the findings raises an information visualization challenge: to produce clear and meaningful visual representations that reinforce human cognition and assist the user to gain understanding about the underlying phenomena and causal relationships suggested by the data [17]. The purpose of using visualization in the proteomics context is to provide an effective mechanism for establishing alternative informative views that can in turn provide biological insight, while abstracting away the details of a large dataset that could be overwhelming to the user.

In this paper, we show that the joint visualization of *meta-features*, along with features emanating from experimental steps, can indicate a powerful mechanism for addressing biological questions and formulating new hypotheses in the context of proteomics analysis. The presented visualizations are generated using the VIP software [18], a user-friendly tool that allows the visual integration and exploration of proteomics data and metadata. Through representative scenarios we highlight and discuss several functionalities of the VIP software that allow the users to: (1) perform the desired graphical encoding according to their needs, (2) control the parameters of the visualization, (3) interact with the visualization, and (4) expand the features workspace by creating new features based on the combination of existing ones. In the following subsections we present and discuss the limitations of several approaches related to proteomics visualization, we provide examples of meta-features, and describe how the proposed visualization can assist in the interpretation of proteomics results.

### Related work

In proteomics tools, we find several visualization attempts for the differential display of proteomics datasets, the representation of LC/MS data sets as "virtual gels", and the annotation of 2D-gel spots. For example, in Proteinscape the 2D gel spots are linked with their identification data and annotated with a colored cross, which is difficult to discern in a crowded 2D gel image, according to the level of identification [19]. Delta2D is an image analysis software that stands out for its impressive differential display based on the spots intensity, and color-coding of the peaks, which highlights proteins that are differentially expressed in specific conditions [20,21]. Label color-coding is also used to illustrate protein properties, such as pI and MW, using continuous color gradients. However, adding large color labels to an already busy 2D gel image creates a visual result that is difficult to process. Pep3D summarizes an LC-MS/MS dataset by placing the peptide peaks in a 2D gel-like image, known as "density plot", using as coordinates the retention time (RT) and mass-to-charge ratio [22]. In Pep3D, the score values of peptide identification and the precursor ions selected for fragmentation are depicted with colored boxes around the peaks. However, Pep3D only allows the visualization of a single experiment at a time, and the boxes used to annotate the peaks are too small to distinguish the color differences. Color has also been used to display the ratio of differential expression levels of identical peptides in two different datasets, in 2D or 3D plots [23,24]. The height and color of cones representing proteins have been used to display up/down regulation [25].

Despite these attempts to visualize either 2DGE-MS or LC-MS/MS data, these tools: (a) exploit either the size or color of the glyph used to encode information and create poor-in-information visual results, and (b) do not allow the combined visualization of features coming from different steps of a proteomics analysis. In the meantime, the integration of any user-selected proteomic features, including metadata, into interactive visual representations, remains an open problem and poses insightful challenges in bioinformatics research.

### Definition of meta-features

The goal of proteomics is to "capture" the proteome at a specific biological state and study the differential expression, functionality, interactions, and post-translational modifications of the proteins. Thus, it is common practice for proteomics researchers to correlate the experimental findings with knowledge gained from the literature and data stored in frequently updated databases, such as the protein type, biological process, location in the cell, molecular pathways and more.

In particular, the protein types characterize groups of proteins that have similar functionality (e.g., the enzymes that catalyze chemical reactions). *Protein functionality *refers to the activities (e.g., catalytic activity, transporter activity, binding) that can be performed by proteins. A series of such activities (i.e., functions) specify a *biological process *(e.g., biosynthetic process, signal transduction, gluconeogenesis). Additionally, the *protein location *(e.g., nucleus, cytoplasm, mitochondrion) is a particular cellular compartment where the protein is known to play its active role.

A *protein network *is a graph modelling protein interactions: the nodes represent proteins and the edges direct or indirect interactions between them [26]. Protein networks are widely used to summarize experimental results, to infer unknown functions of proteins and to shed light on complicated molecular mechanisms. On the other hand, *biological pathways *are subsets of networks containing proteins that communicate a signal from one part of the cell to another during a biological process. *Post-translational modifications *(PTMs) are chemical modifications that take place after the protein translation [27,28]. In particular, PTMs can affect folding, increase or decrease protein activity, and alter protein functionality. Therefore it is likely that PTMs reveal proteins related to observed phenotypical differences. The identification of PTMs (e.g., phosphorylation, acetylation) is important because it can provide insight on the function and role of proteins in biological systems.

Other meta-features can be considered as well, such as protein-drug correlation, structural information, literature references and others, that might indicate even more motivating questions.

### Visualization of meta-features

In previous work, we have introduced Proteomic Feature Maps (PFMs) [7], a novel visualization approach that represents proteomic objects (i.e., proteins or peptides) as spheres, and encodes any two user-selected proteomic features using its size and color. Two more features are also encoded to the spheres (x, y)-coordinates on a map. We have also developed VIP (Visualization for Integrated Proteomics), a user-friendly software tool for the visual exploration and analysis of heterogeneous proteomics datasets based on the PFMs concept [18]. VIP (1) *integrates *proteomic features, (2) *combines *them visually to form PFMs and (3) offers several *filtering*, *navigation *and *interaction *capabilities to the users. The flexibility of the PFMs visualization methodology supported by VIP allows creating PFMs using any desired proteomic features, thus providing a mechanism for generating easily different perspectives for a proteomics experiment. More details on the software are provided in the *Methods *section.

Meta-features in PFMs scenarios were not included in our previous work [7]. However, it is a major task to be considered since meta-features allow addressing challenging biological questions, such as: "*Are the identified enzymes differentially expressed?*", "*Which functions are associated with the up regulated proteins?*", "*Which proteins, among the differentially expressed ones, have also undergone post-translational modifications*?". Importantly, PFMs visualization is intentionally grounded on two-dimensional maps that resemble 2D-gels, a familiar data representation in proteomics, only now enriched with color and size cues defined by the user. This simple idea of augmenting existing representations [29,30] makes the PFMs an intuitive and useful tool for proteomics practitioners.

We demonstrate the effectiveness of the proposed visualization methodology through three indicative proteomics scenarios, where appropriately designed PFMs are used to provide visual answers to important questions, meaningful in the context of proteomics data analysis and interpretation. For each presented scenario, we demonstrate the use of the proposed visualization methodology to generate visual summaries that can effectively address these questions. In the discussion of each scenario, we also highlight several interaction and navigation functionalities of the software. Table 1 provides a summary of the scenarios, the questions addressed and the coded names of the corresponding PFMs. The presented examples aim at showing the flexibility of the approach and the VIP software; other meta-features could also be used to generate user-specific PFMs.

**Table 1:** Case studies summary

	CASE STUDY 1	CASE STUDY 2	CASE STUDY 3
**DESCRIPTION**	Involves the proteins participation in interaction networks and pathways.	Combines the differentially expressed proteins with basic meta-features (e.g., protein type, protein location, mol. function)	Associates the proteins with discovered post-translational modifications (PTMs)

**QUESTIONS** **ADDRESSED**	Q1: Which are the up/down regulated proteins that participate in the network *N*?	Q1: Which proteins were found to be differentially expressed based in both the *X *and *Y *iTRAQ ratios?	Q1: Are the *phosphorylated *proteins also differentially expressed?
	Q2: What are the molecular functions of the proteins that belong to network *N*?	Q2: Are there any upregulated proteins found only in the *X *or in the *Y *iTRAQ ratio?	Q2: Which proteins have undergone *phosphorylation*?
	Q3: What types of proteins (e.g., enzymes, transporters) are involved in the network *N*?	Q3: What is the location of the differentially expressed proteins?	Q3: What is the peptide sequence that is "responsible" for a *phosphorylated *protein?
	Q4: What types of proteins appear in pathway *P*?	Q4: What types of proteins (e.g., enzymes, transporters) are up-regulated?	Q4: Do the proteins that have undergone *oxidation *belong to any network/pathway?
	Q5: Which molecular functions/biological processes are assigned to the proteins that belong to pathway *P*?	Q5: What are the molecular functions/biological processes associated with the up-regulated proteins?	Q5: What is the function of the proteins that have undergone *acetylation*?
	Q6: Are there any common proteins in pathways *P1 *and *P2*?		

**PFMs** **USED**	Network Map PFM=[belongs to network N, fold change]	Differential Expression Comparison Map PFM=[iTRAQ ratios comparison state, iTRAQ differential expression]	Phosphorylation Map PFM=[has undergone phosphorylation, iTRAQ ratio]
			
	Pathway Map PFM=[belongs to pathway P, protein type]		

A summary of the scenarios presented and discussed in the paper: short description, real-life questions concerning each scenario and the corresponding PFMs. The notation *PFM = [feature1, feature2] *is used to show quickly the two features that have been associated with the size and color of the spheres respectively.

The scenarios we chose to present show that VIP can be used to address effectively questions that biologists commonly ask during a proteomics analysis. These real-life questions, based on meta-features (e.g., protein type, post-translational modifications, involvement in pathways and biological networks), were revealed from our close interaction with a group of scientists in the Biomedical Research Foundation of the Academy of Athens [29], who use proteomics routinely in order to elucidate biological mechanisms.

By applying the PFMs visualization to real-life scenarios we show that VIP users with minor effort can:

• *Integrate visually *disparate proteomic features originated from different databases and tools.

• Create *multiple views *for a proteomics experiment in order to explore a dataset from alternative perspectives.

• *Query*, *navigate *and *interact *with their visualizations, in order to retrieve useful knowledge by recognizing patterns and correlations.

• *Skip the time-consuming task *of working on long protein lists when looking for biologically relevant relations suggested by their results.

## Results and Discussion

In this section, we present three representative scenarios that demonstrate the ways in which our visualization methodology can be used to accelerate and enhance proteomic analysis. Each scenario exercises a different set of meta-features that can be visually combined and integrated using the VIP software tool. Moreover, for each scenario we discuss how VIP can help the users interact with the visualization and address specific questions.

### Scenario 1 - Networks and Pathways Features

This scenario demonstrates how VIP contributes to data interpretation by facilitating the association of proteomics experimental results with information related to biological networks and pathways.

As biology is transformed into an information-driven integrative science, it becomes increasingly common, captured during VIP requirements analysis, to correlate experimental findings with known protein interaction networks and pathways, in order to infer the underlying biological mechanisms suggested by the data [8-13]. Moreover, in graphs and diagrams used to visualize pathways and networks, it is typical to include information regarding the protein differential expression (e.g., fold change), protein type, protein name, molecular function and more.

We present below six typical questions, regarding the involvement of proteins in biological networks and pathways, which will be addressed visually by the VIP tool and the PFMs visualization.

Q1: Which are the up/down regulated proteins that participate in the network "Cellular Assembly and Organization, Neurological Disease, Small Molecule Biochemistry"?

Q2: What are the molecular functions of the proteins that belong to the network "Lipid Metabolism - Small Molecule Biochemistry - Cell Cycle"?

Q3: What types of proteins (e.g., enzymes, transporters, cytokines) are involved in the network "Cell Morphology - Cell Cycle - Inflammatory Response"?

Q4: What types of proteins appear in pathway "Glycolysis/Gluconeogenesis"?

Q5: Which molecular functions/biological processes are assigned to the proteins that belong to pathway "Pentose Phosphate"?

Q6: Are there any common proteins in pathways "Fatty Acid Metabolism" and "Glycolysis/Gluconeogenesis"?

In the next two subsections we detail two typical uses of the PFMs visualization in this context. The corresponding PFMs were based on the dataset generated from a 2DGE-MS proteomics experiment (described in *Methods*).

### Network Map

**Figure 1:**
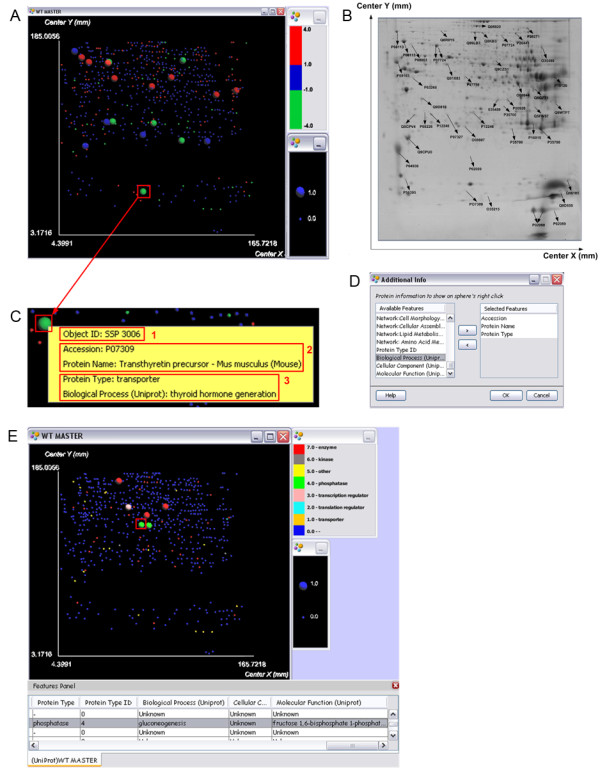
**Network and pathway maps of a 2DGE-MS experiment**. (A) In the network map of Scenario 1, large spheres indicate proteins that belong to a specific network, whereas their color (red/green/blue) indicates proteins' up/down/no regulation. (B) A 2D gel image annotated with the accession numbers of selected identified proteins. Such images, very often found in proteomics publications, are difficult for the user to explore and need effort to indicate the interesting proteins/spots. The maps presented in Scenario 1 use the detected spots of this 2D gel. (C) The result of a right click action on a specific sphere is a pop-up menu that contains user-selected information. In this case, the menu shows the Object ID, Accession Number, Protein Name, Protein Type and Biological Process of a protein. (D) The user can select any one of the available features to appear in the pop up menus of the spheres. (E) In the pathway map of Scenario 1, large spheres indicate proteins that belong to a specific pathway, whereas their color (red/green/blue) indicates their protein type (also shown in the color bar). The result of a click action on a specific sphere is highlighted corresponding table row, which contains all the features of the clicked sphere/protein.

In the PFM of Figure 1A (network map), we visualize jointly the binary feature "protein belongs to network Cellular Assembly and Organization, Neurological Disease, Small Molecule Biochemistry" and the volume fold change feature, which shows the differential expression (i.e., up/down regulation) of proteins (see *Methods*). Specifically, in the PFM of Figure 1A, large spheres represent proteins that participate in the network, while red/green spheres are up/down regulated protein spots (i.e., having log fold change value larger/smaller or equal to 1/-1). Spots that had log fold change in the range [-1,1] are depicted as blue spheres.

A brief examination of this PFM provides a visual impression of the up/down regulated proteins of the experiment that also participate in the specific network. Moreover, the proteins that belong to the network but were not differentially expressed are also easily visible as large blue spheres. As a result, this network map summarizes effectively the proteins of a dataset that belong to an interaction network, in conjunction with their differential expression information. The network map does not aim at replacing network graphs, which demonstrate the interactions of all proteins involved in a biological network. Instead, the objective of this network map is to visualize whether the proteins identified in a certain experiment belong to a network of interest or not, while preserving a visual reference to the familiar 2D gel images of the experiment (Figure 1B). The PFM visualization also provides a clear, easy to grasp and interpret image when compared to a manually annotated gel image, which is usually crowded with spot ids and accession numbers (Figure 1B). Such annotated gel images, which are commonly found in published 2DGE-based proteomics studies [11,30,31], tend to be cluttered with arrows, circles and underlined font to mark differentially expressed spots, making it even trickier for the reader to distinguish interesting information.

The interaction capabilities offered by VIP can be exploited in order to address questions similar to those described before. For example, clicking on the sphere shows the Object ID of a protein (Figure 1C1), which characterizes uniquely every proteomic object in the VIP workspace and corresponds to the spot number of the protein in the gel. Thus, the user can answer a question similar to Q1: "SSP 3006 is a down regulated protein that participates in the network Cellular Assembly and Organization, Neurological Disease, Small Molecule Biochemistry". To answer the same question in terms of the accession number or the name of a protein, the user simply has to include these features in the pop-up menu of the sphere. It then becomes clear that the selected green and large sphere, which corresponds to a down regulated protein involved in the network, has been identified by the accession number "P07309" and is known as "Transthyretin precursor - Mus musculus (Mouse)" (Figure 1C2).

Appending features to the pop-up menu of the spheres (Figure 1D) is straightforward and allows handling even more questions, such as Q2 and Q3, which involve the protein type and molecular function. Figure 1C3, shows that the selected down regulated protein that belongs to the specific network acts as a "transporter" and is associated with the molecular function "hormone activity". This useful VIP attribute allows the user to retrieve the desired information for the proteins, while exploring the map.

By creating a network map using the VIP tool, the users benefit from:

(a) The PFM visualization as a means to summarize the proteins participation in an interaction network, along with their up/down regulation information.

(b) The VIP feature to rapidly access specific feature values, and create quick visual displays in order to address network-related questions.

### Pathway Map

In the PFM of Figure 1E (pathway map), we visualize jointly the binary feature "protein belongs to the pathway Glycolysis/Gluconeogenesis" and the protein type (e.g., enzyme, transporter) (see *Methods*).

A quick examination of the pathway map reveals proteins that belong to the pathway of interest among the proteins identified by the analysis (large spheres), as well as the different types of proteins found to participate in the pathway (three different colors of the large-size spheres: red, green and pink). The protein type (question Q4) can be retrieved from the color of the sphere, the pop-up menu, or the highlighted row of the table that stores all features of the protein (Figure 1E). Using the table also gives the advantage to inspect all features related to the selected protein-sphere. For example, one can retrieve from the table the molecular functions biological processes associated with the proteins that belong to the pathway (question Q5).

Furthermore, to deal with a question similar to Q6, VIP offers the capability to produce new features based on existing ones and expand the features workspace on demand. For example, several "belongs to pathway" binary features (see *Methods*) can be added to produce a new counter-type feature that records the number of pathways an identified protein belongs to. This new feature could then be visualized on a new PFM in order to distinguish proteins that are found uniquely in a specific pathway from proteins that participate in several pathways.

To summarize, a pathway map, constructed using the VIP tool, can facilitate the interpretation of proteomics analysis results by offering:

(a) The effortless discrimination of proteins that participate in a pathway along with significant complementary relevant features, such as the protein type and molecular function.

(b) The capability to create on demand new features based on existing ones in order to expand the features workspace and the perspectives under which a proteomics experiment can be visually explored.

### Scenario 2 - Differential Expression Features

This scenario shows how the proposed visualization methodology and the functionality of the VIP software can enhance the differential expression analysis part of a proteomics workflow.

Importantly, quite often researchers want to associate the differentially expressed proteins of a proteomics dataset with different protein-related meta-features. Such features, obtained by the GO, the Ingenuity Pathway Analysis or other sources, may include (but are not limited to):

• Protein type (e.g., enzyme, kinase, transporter, transcription regulator, peptidase)

• Protein location (e.g., cytoplasm, nucleus, extracellular space, plasma membrane)

• Molecular function (e.g., actin binding, kinase activity, protein binding)

• Biological Process (e.g., anti-apoptosis, cellular biosynthetic process, DNA replication)

We present below five representative proteomic questions relevant to this scenario that will be addressed visually in the next subsection.

Q1: Which proteins were found to be differentially expressed based in both the 118/116 and 119/117 iTRAQ ratios?

Q2: Are there any up regulated proteins found only in the 118/116 ratio or in the 119/117 iTRAQ ratio?

Q3: What is the cellular location of the differentially expressed proteins?

Q4: What types of proteins are up regulated?

Q5: What are the molecular functions/biological processes associated with the up regulated proteins?

The map described in the following paragraph was based on a dataset from a LC-MS proteomics experiment (see *Methods*).

### Differential Expression Comparison Map

**Figure 2:**
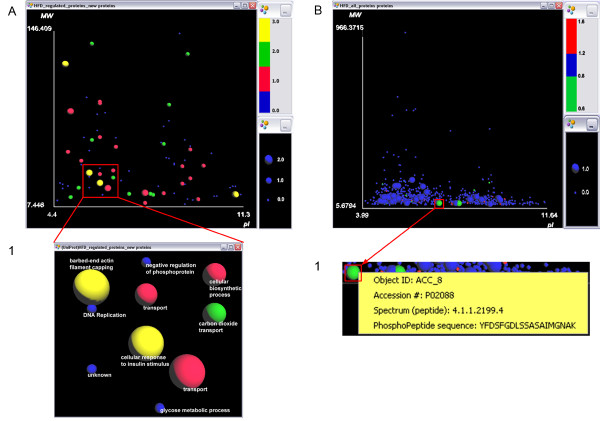
**Differential expression and phosphorylation maps of a LC-MS/MS experiment**. (A) The differential expression map of Scenario 2 shows a subset of proteins identified by the LC-MS/MS experiment. The size indicates if the proteins are uniquely (medium size) or commonly differentially expressed (large size) in two pairs of biological states (i.e., in two iTRAQ ratios). The color indicates the type of differential expression: red (green) for up (down) regulation in at least one ratio, yellow for up/down regulation in the two ratios, and blue for no differential expression in any ratio. (A1) Zoomed area of the differential expression comparison map, showing the Biological Process associated with each protein in the spheres labels. (B) The phosphorylation map of Scenario 3 shows all proteins identified by the LC-MS/MS experiment. Large spheres indicate proteins that have undergone phosphorylation, whereas their color (red/green/blue) indicates their up/down/no regulation based on an iTRAQ ratio. (B1) Pop up menu of a right clicked protein, showing its Accession Number, the Spectrum id and the sequence of the corresponding phosphorylated peptide.

To create the differential expression comparison map (Figure 2A), we defined the feature "iTRAQ ratios comparison state" (see *Methods*) that allows locating easily the differentially expressed proteins in one or both iTRAQ ratios. The ratios we used (i.e., 118/116 and 119/117) are important for the specific study since they capture differences within the wild type and transgenic mice respectively, when the mice are fed with normal diet (i.e., numerator labels 118 and 119) or injected with an anorectic toxin (i.e., denominator labels 116 and 117). Thus, each iTRAQ ratio is indicative of the differential expression between two biological states. The "iTRAQ ratios comparison state" is used to indicate proteins that fall in one of the three categories:

S0: not differentially expressed in any of the two ratios.

S1: differentially expressed in one of the two ratios only (i.e., either in wild type or in transgenic mice).

S2: differentially expressed in both ratios (i.e., in both types of mice).

For simplicity, we will call the 118/116 and 119/117 iTRAQ ratios, as Ratio 1 and Ratio 2. A quick examination of the differential expression comparison map (Figure 2A) reveals the proteins that have been differentially expressed in one or both iTRAQ pairs (i.e., medium and large spheres respectively) (question Q1). Proteins without significant change in their expression levels are depicted as small spheres. It is important to note that this map shows only a subset of the identified proteins (i.e., 77 out of 691 proteins), which were differentially expressed at least in one of the iTRAQ ratios used. This user requirement reflects the emphasis given on the proteins expression in a differential proteomics study.

We also created the feature "iTRAQ differential expression" (see *Methods*) in order to represent the direction of differential expression (i.e., up or down regulation) in each one of the two ratios. For example, if a protein is associated with the "up/down" value it means that this protein was found to be up regulated in Ratio 1, and down regulated in Ratio 2. Similarly, the "down/down" value indicates down regulation in both ratios, while the "-/up" value suggests that the protein was up regulated in Ratio 2 only. After taking into consideration these user requirements, the following categories related to this feature were formed:

C0: non differentially expressed proteins (blue), C1: up regulated proteins, at least in one ratio (red),

C2: down regulated proteins, at least in one ratio (green),

C3: up regulated proteins in one ratio and down regulated proteins in the other (yellow).

Table 2 summarizes and explains the size and color categories used for the spheres of this map. The common use of size and color to encode differential expression information, also known as redundant encoding [32], was deliberately chosen in order to provide quick and reliable perception of the important features of differential expression. To answer a question similar to Q2, the user has to simply observe the map and look for the spheres that are of medium size (proteins differentially expressed only in one ratio) and red (proteins up regulated).

**Table 2:** Size and Color categories in Differential Expression Comparison map

		SIZE CATEGORIES		
**COLOR** **CATEGORIES**		**S0** **(small)**	**S1** **(medium)**	**S2** **(large)**
	
	**C0** **(BLUE)**	**-/-**	NA	NA
	
	**C1** **(RED)**	NA	**UP/-** **-/UP**	**UP/UP**
	
	**C2** **(GREEN)**	NA	**DOWN/-** **-/DOWN**	**DOWN/** **DOWN**
	
	**C3** **(YELLOW)**	NA	NA	**UP/** **DOWN/** **DOWN/** **UP**

The table summarizes the size and color categories used for the Differential Expression Comparison map, as well as the values assigned to each category. The X/Y annotation is used to indicate that X (Y) is the type of differential expression in Ratio 1 (Ratio 2) respectively, whereas the dash indicates no differential expression in the corresponding category (e.g., UP/- means up regulation in Ratio 1 and no significant differential expression in Ratio 2). NA denotes a not available combination of size and color.

From our interaction with the research group that carried out the experiment and produced the dataset, we noticed that the users found it difficult to correlate the two ratios, and to identify the proteins of interest by looking into a large table (i.e., spreadsheet) with many iTRAQ numbers. However, they responded very positively in the combined visualization of the iTRAQ ratios and integrated it into their workflow because they were able to locate without effort the proteins that are differentially expressed in only one ratio, or in both ratios.

This map also offers visual connection of the differential expression proteins with important protein-related meta-features, such as the protein location, protein type, as well as GO classification. In particular, the user could map the protein location (question Q3), protein type (question Q4), molecular function or biological process (question Q5) to the label of the spheres in order to get immediate access to the values of these features. For example, in Figure 2A1 each protein-sphere carries a label that denotes its biological process obtained from the GO.

To sum up, the differential expression comparison map, created using the VIP tool, allows the user to:

(a) Find and represent visually the proteins that were differentially expressed in one or more biological states of interest.

(b) Have instant access to the values of a selected feature using the label of the spheres.

### Scenario 3 - Post-translational Modification Features

In this scenario we demonstrate how the proposed visualization methodology can assist the users explore visually the post-translational modifications (PTMs) that exist in a proteomics dataset.

The increasing importance of detecting protein modifications when studying phenotypes [27,33-35], motivated us to evaluate the usefulness of PMFs based visualization in a study concerned with PTMs. PTMs are known to alter the protein properties, such as their molecular function, interactions with other proteins, and participation in a biological pathway. The discovery of a PTM also provides strong motivation to a biochemist to look deeper into the protein sequence and identify the modified peptide. Once the modified peptide is identified, one can search whether this modification is already known, or it is a new one. If a known modification has occurred, interesting assumptions can be made for the function of the proteins and their possible role in certain pathways. If the detected modification is unknown, then a new series of experiments can be designed in order to study the importance and role of the PTM in the function and activity of a protein.

We present below five interesting questions regarding protein PTMs that can trigger the researcher to perform further investigation on the modified proteins. The following subsection also describes how PFMs based visualization and the VIP tool can help the user address these questions.

Q1: Are the phosphorylated proteins also differentially expressed?

Q2: Which proteins have undergone phosphorylation?

Q3: What is the peptide sequence that has undergone phosphorylation?

Q4: Do the proteins that have undergone oxidation belong to a specific network/pathway?

Q5: What is the function of the proteins that have undergone acetylation?

The map described in this scenario was also based on the LC-MS dataset (described in *Methods*).

### Phosphorylation Map

For the phosphorylation map of Figure 2B, we used the binary feature "protein has undergone phosphorylation" and the 118/116 iTRAQ ratio, which shows the differential expression (i.e., up/down regulation) of proteins (see *Methods*). The experimental indication about the phosphorylation of the proteins was based on the results of the ProteinPilot software [36] used in this study. This map shows all identified proteins of the experiment (i.e., 691 proteins, as opposed to the map of Scenario 2) in order to include not only *all *differentially expressed, but also *all *phosphorylated proteins.

The phosphorylation map of Figure 2B visually confirms that apart from the differentially expressed proteins, one should also put particular emphasis on the identification of PTMs: from the proteins that have undergone phosphorylation (i.e., large spheres), only two have been differentially expressed (i.e., large and green spheres) (question Q1). This finding shows that if the researchers had restricted the analysis only to the up/down-regulated proteins, they would have missed the significant information that the post-translationally modified proteins carry (i.e., large blue spheres).

This map offers an effective visual summary of the phosphorylated proteins that have been detected within the experiment. It also assists in identifying proteins that have undergone a PTM by retrieving their accession numbers (question Q2). Moreover, if this information is available and imported in the VIP workspace, the user could also retrieve the sequence of the modified peptide of a phosphorylated protein, as well as the peptide spectrum id (Figure 2B1). Using the spectrum id, further analysis can be performed, such as viewing the mass spectra file in the corresponding software (e.g., ProteinPilot [36]), in order to verify the modified peptide.

The user can also combine the information regarding the phosphorylated proteins with pathway or network-related meta-features, as well as the molecular function to address questions similar to Q4 and Q5. Due to space limitations we do not provide examples using these features, but we believe that the examples presented so far have provided convincing evidence on the flexible adaptation of PFMs based visualization to the proteomics analysis specific objectives.

Using a phosphorylation map, or a map regarding any PTM (e.g., oxidation, methylation etc.), the user can:

(a) Establish a quick visual summary of the proteins that have undergone the specific PTM and retrieve their accession number, sequence and spectrum of the modified peptide in order to perform further analysis.

(b) Combine easily the PTM-related information with other important meta-features (e.g., molecular function, interaction networks, pathways presence) to formulate new hypotheses on the potential role of a protein in certain molecular mechanisms.

## Conclusions

We have demonstrated through three proteomics scenarios, which were defined from different user requirements, that the joint visualization of features, typically produced by a proteomics experiment, along with meta-features, can indicate a powerful mechanism for addressing biological questions and formulating new hypothesis for further investigation.

Throughout the discussion we have pointed out some of the most significant functionalities of the VIP software that can provide effective and comprehensive exploration of the PFMs. In particular, the users can with minor effort: (1) perform the desired graphical encoding according to their needs, (2) control the parameters of the visualization, such as the values-to-colors association, (3) interact with the PFMs in order to rapidly retrieve specific feature values of the displayed proteomic objects, and (4) expand the features workspace by creating new features by combining existing ones.

In summary, the PFMs visualization, offered by the freely available and user-friendly VIP software, allows the users in the field of proteomics to:

*• Visually integrate *unconnected proteomic features coming from different meta-analysis sources (i.e., databases and pathway/network analysis tools).

*• Generate alternative views *for a proteomics experiment, in order to analyze and explore a heterogeneous dataset from *multiple perspectives *according to their needs and objectives.

*• Query, navigate and interact *with their data and the produced visualizations in order to address visually the biological questions raised in a proteomics analysis context.

*• Avoid the time-consuming and error-prone task *of looking for correlations and interesting relations within large tables of raw data.

Due to its data integrative nature, the described approach and associated software tool have the potential to address major challenges in proteomics data analysis and the fast growing discipline of systems biology. For example, the differential comparison of biological conditions that is also supported by VIP (e.g., through the differential display of multiple PFMs), as well as the capability to simultaneously display and compare on a map multiple features, can facilitate inspecting biological system properties at a global scale. Although the methodology has been developed for proteomics it can be applied to any system with components that can be modelled by a set of features.

The evaluation of the usability of the software is also in progress, using a task-driven methodology targeted to the needs of proteomics practitioners. This evaluation will help us examine the users' reactions to specific tasks (e.g., the creation of new features), enhance the proposed visualization methodology, and possibly expand the functionality of the software. To the best of our knowledge, VIP is currently the only software tool available in the public domain that supports exploration-by-visualization of large-scale heterogeneous proteomics datasets combining data and meta-data.

## Methods

### Datasets

This section provides information on the datasets used in the presented scenarios. Experimental details that are irrelevant to the work described in the paper are not provided.

The objective of the 2DGE-MS study (dataset used in Scenario 1) is the identification of proteins involved in the mechanisms that lead to the development of fatty liver in mice. In this study, wild type and transgenic mice were fed with high fat diet (i.e., an experimental manipulation to induce obesity or even to unmask related phenotypes in mice) or normal diet (disease and control groups respectively) and resulted in 4 categories: (1) Wild type with normal diet, (2) Wild type with high fat diet, (3) Transgenic with normal diet and (4) Transgenic with high fat diet. Liver tissues from all experimental groups were subjected to proteomics analysis creating four 2DGE gels per category. Pairs of categories were compared and in each comparison the subset of matched spots that passed at least two of the three statistical tests used (i.e., t-test, Mann-Whitney, Partial Least Squares) and also satisfied the volume 2-fold change quantitative criterion were considered as differentially expressed. The differentially expressed spots in the diseased versus the healthy subjects were then identified using peptide mass fingerprinting (PMF). The PDQuest image analysis software [37] was used for spot detection and matching and MASCOT identification engine [38] for protein identification.

The LC-MS study (dataset used in Scenarios 2 and 3) aimed at identifying the differentially expressed proteins in a well-characterized mouse model of high fat diet induced obesity, as well as in a model of lipopolysaccharide (LPS) induced anorexia. In this study, both wild type and transgenic mice were fed with high fat diet or normal diet, or were injected with an anorectic dose of LPS. The quantitative LC-MS/MS based method used 8-plex iTRAQ™ reagents and resulted in several categories designated with the 116, 117, 118, 119 and 121 reporter ions. Each category corresponds to a different state (e.g., 116: Transgenic mice with normal diet, 117: Wild type with normal diet, 118: Transgenic mice with LPS, 119: Wild type with LPS, 121: Wild type with high fat diet). The reporter ions ratio (e.g., 118/116, 119/117) is indicative of the differential expression between the two biological states of interest. In Scenario 2, we used two ratios to capture differences between the transgenic mice with normal diet and LPS (118/116), and the wild type mice with normal diet and LPS (119/117). Finally, the proteins were identified using MS/MS and the ProteinPilot software [36].

### Meta-Features

For the studies already described, meta-analysis was performed using the Ingenuity Pathway Analysis software package [39], which provided us with the protein type and location, a list of interaction networks and canonical pathways and many more meta-features. We also exploited the VIP capability to perform an online query search to UniProt database [40] and retrieve for each protein additional meta-features, such as the number of amino acids (AA), the theoretical isoelectric point (pI) and molecular weight (MW), the grand average hydrophobicity index (GRAVY), and the Gene Ontology annotation, including the Biological Process (BP), Molecular Function (MF) and Cellular Component (CC). Thus, we created a large pool of meta-features, in a simple tab-delimited text format, so as to produce PFMs that could cope with questions similar to those described in Table 1. Moreover, we consider as meta-features the post-translational modifications (PTMs) discovered by advanced high-throughput proteomics technologies (i.e., tandem mass spectrometry-MS/MS), because although they are obtained at the experimental step of mass spectrometry, they can only be sufficiently exploited at a later meta-analysis step in the workflow. The features "belongs to network N" and "belongs to pathway P" of Scenario 1 and the feature "has undergone phosphorylation" of Scenario 2 are called *binary *because they can only have two different values, 1 (true) or 0 (false). Thus, they indicate: (1) the presence or absence of a protein in network N, or pathway P, respectively, or (2) whether a protein has undergone phosphorylation or not. The network/pathway-related features have been created manually by processing the network/pathway lists that the Ingenuity Pathway Analysis software produced. Similarly, we created the PTM-related feature, by processing the proteins list that was exported from ProteinPilot™.

Finally, the feature "iTRAQ ratios comparison state" used in Scenario 2 has been created using the VIP capability to compute new features, either by performing a *function rule*, or by adding existing features (i.e., *sum rule*). To produce this feature we followed the steps:

1. Create a temporary feature, based on the function rule:

    IF ((Ratio1 < 0.8) OR (Ratio1 >1.2))

                temp_feature1 = 1

    ELSE

                temp_ feature1 = 0

This temporary feature assigns the value 1 to the proteins that are either down (i.e., Ratio1<0.8) or up regulated (i.e., Ratio1>1.2).

2. Similarly, produce another temporary feature, using the same rule but for Ratio2.

3. Use the sum rule to add the values of the two temporary features. The result is the feature "iTRAQ ratios comparison state", which has three distinct values: 0, 1 and 2, denoting the number of ratios that a protein was found to be differentially expressed on.

### Graphical encoding

We use the size of the spheres to visually encode the binary features (i.e., 1/0 for true/false); small and large spheres show easily the two different states of the feature. For example, large spheres indicate proteins that participate in a network or a pathway (Scenario 1), or proteins that were phosphorylated (Scenario 3). Small spheres depict the rest of the proteins in the dataset (Scenario 1), or proteins that were not phosphorylated (Scenario 3).

On the other hand, color was exploited to encode features with values in a discrete or continuous range. For example, in the network map of Scenario 1 (Figure 1A) we use color to represent the fold change feature in order to preserve the familiar association of color with differential expression (up regulation - red, down regulation - green) adopted since the early microarray-based genomic studies. In the pathway map of Scenario 1 (Figure 1E), we also used color to encode eight different protein types. Since protein type is a categorical feature, we first associated each protein type with a number (e.g., transporter: 1, translation regulator: 2). We also created a user-defined color map and assigned different colors to the protein types, so as to distinguish easily proteins of the same type (e.g., red: enzymes, orange: transporters). The color and protein type association is shown in the color bar of Figure 1E.

In Scenario 2, we encoded the three discrete values of the "iTRAQ ratios comparison state" feature (0 for category S0, 1 for category S1 and 2 for category S2) to size. Thus, three easy-to-grasp size categories were shaped: small, medium and large spheres respectively. For the "iTRAQ differential expression" feature, we used 4 different colors to depict the four different categories: blue for non-differentially expressed proteins, red for up-regulated, green for down regulated, and yellow for proteins up-regulated in one ratio and down-regulated in the other.

In Scenario 3, we chose to encode the 118/116 iTRAQ ratio to color, in order to visualize the up/down regulated proteins as red/green spheres (i.e., having 118/116 ratio > 1.2 or < 0.8 respectively) and the proteins with no significant differential expression change (i.e., 118/116 ratio between 0.8 and 1.2) as blue spheres.

In general, for features with a small number of distinct values (e.g., up to 5 values), size can be employed to depict differences. In contrast, color is well suited to be used for features with a larger number of values, or categorical features. Additionally, color can also be a suitable encoding choice for continuous values (e.g., by changing the lightness of a color), or for distinguishing conditions (e.g., up regulation - red, down regulation - green).

### Proteomic Feature Maps and the VIP software

PFMs based visualization is a simple and powerful approach, applicable to any proteomics analysis workflow [7]. PFMs are useful to visualize simultaneously multiple features for the proteins identified in a proteomics analysis. The approach suggests representing proteomic feature sets (including numerical or categorical features) in gel-resembling synthetic maps, by exploiting the x and y coordinates, size, color and label attributes of the spheres.

In previous work [7], we presented a prototype implementation of PFMs using OpenDX [41], an open source visualization software package based on IBM's Visualization Data Explorer. OpenDX was the best choice for a rapid proof of concept of our proposed approach at the time, due to the simplicity of its modular visualization environment. The prototype provided a control panel, which allowed the user to import the features and control the colors assigned to a feature, as well as basic interaction capabilities (e.g., zooming, 3D rotation).

However, in order to create a powerful, stand-alone and user-friendly application for integrative proteomics data visualization, we implemented the VIP software, which supports the PFMs concept. Although the underlying PFMs approach is the same with the OpenDX-based implementation, VIP offers a lot more visualization options and interaction capabilities with the features workspace and the produced visualization, as well as user control on the graphical encoding. In particular, in VIP the user can control several parameters of the visualization through an intuitive interface, such as the number of sphere sizes to be used, the level of transparency of the spheres, and the background of the map. Visual queries can also be performed on the data, in order to filter the visualization results based on a user-defined criterion (e.g., proteins with sequence coverage > 80%). The proteins-spheres that satisfy the given condition get elevated and detached from the map's level, enabling their easy visual exploration. Additionally, VIP supports the interaction between the visualization and the features workspace, allowing the user to explore all proteomic features that are associated with a protein. Finally, the interaction between multiple PFMs is supported allowing the visual comparison of spheres representing the same proteins across different maps. Although some of these capabilities were not described in detail in this paper, they can be easily explored using the provided data samples after installing the software. VIP is implemented using the Java platform (Sun JDK 1.6) and released under the GNU General Public License (GPL). The software has been tested under Microsoft Windows XP and Vista, Mac OS X and GNU/Linux.

The backend of VIP, which is responsible for integrating proteomic features, is based on POML (Proteomics Object Markup Language), our proposed markup language [18]. In particular, we used the Java API for XML Processing (JAXP), an open source API for efficient XML validation and parsing [42].

The PFMs visualization in VIP is based on the Java 3D API [43]. Java 3D is a powerful visualization choice because it offers the advantage of fast application development. In particular, it incorporates a high-level scene-graph model and allows developers to focus on the objects and the scene composition. Importantly, Java 3D takes advantage of the graphics hardware in a system, since it runs on top of either OpenGL or Direct3D technologies. Thus, it achieves high performance by exploiting hardware acceleration and releasing the CPU of the system from drawing complex 3D scenes.

## Competing interests

The authors declare that they have no competing interests.

## Availability and requirements

Project name: VIP

Project home page: http://pelopas.uop.gr/~egian/VIP/index.html

Operating system: Windows

Programming language: Java

Other requirements: JDK 1.7, Java3D 1.5.2

Licence: GNU GPL

Any restrictions to use by non-academics: none.

## Authors' contributions

EGG formulated the scenarios from user requirements, implemented the software used, produced the visualizations and drafted the manuscript. GL assisted in the visualizations creation and revised the manuscript. ESM assisted in the scenarios formation, drafted and revised the manuscript. All authors read and approved the final manuscript.
